# Endogenous retroviruses of the chicken genome

**DOI:** 10.1186/1745-6150-3-9

**Published:** 2008-03-24

**Authors:** Ahsan Huda, Nalini Polavarapu, I King Jordan, John F McDonald

**Affiliations:** 1 School of Biology, Georgia Institute of Technology, Atlanta, GA, USA

## Abstract

We analyzed the chicken (*Gallus gallus*) genome sequence to search for previously uncharacterized endogenous retrovirus (ERV) sequences using *ab initio *and combined evidence approaches. We discovered 11 novel families of ERVs that occupy more than 21 million base pairs, approximately 2%, of the chicken genome. These novel families include a number of recently active full-length elements possessing identical long terminal repeats (LTRs) as well as intact *gag *and *pol *open reading frames. The abundance and diversity of chicken ERVs we discovered underscore the utility of an approach that combines multiple methods for the identification of interspersed repeats in vertebrate genomes.

This article was reviewed by Igor Zhulin and Itai Yanai.

## Findings

Chicken, a modern descendant of the dinosaurs, is the first avian to have its genome sequenced [1]. Phylogenetically, its position between fish and mammals provides valuable insight into the evolution of vertebrates. The chicken genome has a size of 1.2 billion bases, approximately one third of the size of the human genome.

The overall interspersed repeat, *i.e. *transposable element (TE), content of the chicken genome was determined to be less than 9% [1]. This fraction is considerably lower than that of mammalian genomes, where transposable elements (TEs) account for 40–50% of genomic sequences [2-4]. While chicken has long been a model system for the study of retroviruses [5], a mere 1.3% of the chicken genome can be classified as endogenous retroviruses (ERVs) compared to about 5% in humans [3]. Nevertheless, protein coding sequences still make up only a minor fraction of the chicken genome leaving a substantial quotient that has yet to be been accounted for. The authors of the initial analysis of the chicken genome posited that much of the uncharacterized sequence was likely to be derived from unrecognized TEs [1]. Indeed, novel or previously uncharacterized TE sequences may be missed by homology-based methods for the detection of repeats, such as the widely used RepeatMasker program [6], which rely on the comparison of genomic sequences to libraries of known repeat consensus sequences. *Ab initio *methods, on the other hand, identify repeats by virtue of their structural characteristics without regard to any sequence similarity to known elements. We used a combination of *ab initio *detection, sequence similarity searches, motif identification and evaluation of element structural (repeat) features to search for novel ERVs that may have been missed in the initial analysis of the chicken genome.

LTR_STRUC was the first *ab initio *program designed to detect long terminal repeat (LTR) containing elements, such as ERVs, in genomic sequence [7]. Briefly, LTR_STRUC works by sliding a window along genomic sequence and looking for direct repeats that are spaced apart within a specified size range (*e.g. *5–10 kb). After identifying putative LTRs in this way, it searches for other characteristic features of LTR elements such as target site duplications, inverted repeats at LTR termini, primer binding sites and poly purine tracts. Based on these features, it predicts the direction of the LTR element and provides the corresponding three frame translation of the reverse transcriptase (RT) sequence in the internal region of the element. LTR_STRUC has proven effective at identifying novel LTR elements, including ERVs, in chimpanzee [8], mouse [9] and rice genome sequences [10].

LTR_STRUC was run on the 2004 build of the chicken genome sequence, *i.e. *the v1.0 draft assembly from the Washington University Genome Sequencing Center [1] distributed on the UCSC Genome Browser [11], resulting in the detection of 39 putative full-length LTR elements. RT homologous sequences were identified in these elements and used as queries in TBLASTN [12] searches against the chicken genome sequence. The BLAST output and flanking genomic sequences were visually inspected to look for ERV characteristic features such as LTRs, target site repeats and terminal inverted repeats. LTRs are direct repeats at the 5' and 3' termini of the ERVs that are ~200–350 bp in length. Characteristic dinucleotide terminal inverted repeats are found at the beginning (TG) and ends (CA) of ERV LTRs. Target site repeats are short (4–6 bp) direct repeats found immediately upstream and downstream of ERV insertions that result from resolution of a staggered break that is made when the elements integrate in the genome. We identified a total of 89 putative ERVs in the genome using the combined *ab initio*, sequence similarity and element feature detection approach. The presence of intact open reading frames that encode sequences that have significant sequence similarity to RT along with the canonical RT catalytic motif [13] were used to validate 61 of these cases as intact full-length ERVs.

**Figure 1:**
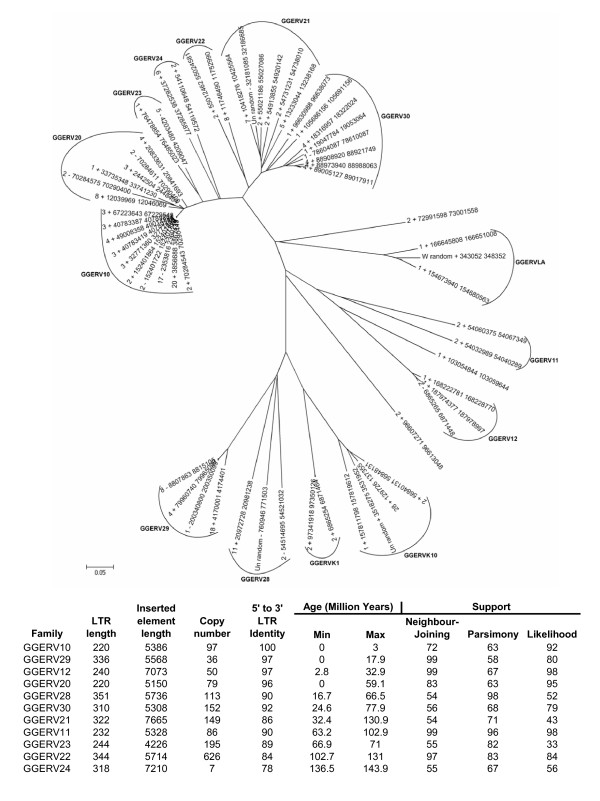
**Chicken endogenous retrovirus families**. Phylogenetic analysis of an RT multiple sequence alignment for all full-length elements was used to delineate chicken ERV families. The neighbor-joining phylogeny is shown; maximum parsimony and maximum likelihood trees were also reconstructed. The names of the taxa (ERV sequences) correspond to the chicken chromosome number, strand, start and end coordinates from the May 2006 build, v2.1 draft assembly from the Washington University Genome Sequencing Center, found on the UCSC Genome Browser. Family names and characteristics for the 11 novel ERV families discovered here are shown below the tree. Family copy numbers are indicated along with the family averages of intra-element percent identity between 5' and 3' LTRs and their age ranges (lower-to-upper bounds). For each family, percent support values are shown for the internal branch that subtends the family based on bootstrap analysis, for neighbor-joining and maximum parsimony, and the approximate likelihood ratio test for maximum likelihood.

Phylogenetic analysis of an RT nucleotide sequence alignment was used to classify the chicken ERV sequences that we identified. ERV phylogenies were built using the neighbor-joining and maximum parsimony methods implemented in the program MEGA [14] and maximum likelihood using the program PhyML [15]. For neighbor-joining and maximum parsimony 1,000 bootstrap replicates were run to assess the support for internal branches on the phylogeny, and the approximate likelihood ratio test [16] was used to evaluate the support for branches along the maximum likelihood tree. The ERV phylogeny shows a number of well resolved groups that correspond for 14 distinct families of chicken ERVs, 11 of which are described here for the first time (Figure 1). In the absence of a standard naming convention for viral families in the chicken genome, we named the families using GGERVNN, for *Gallus gallus *endogenous retrovirus followed by the family number. We also reported the new families to Repbase [17] where they constitute nearly half (8 out of 17) of all the ERV families known for the chicken genome.

The 11 new ERV families we discovered using LTR_STRUC and BLAST analysis include 48 full-length elements and 1,542 fragmented sequences, most of which are solo LTRs that result from intra-element LTR-LTR recombination. When representatives of the 11 novel families were used to search the chicken genome for homologous sequences using the RepeatMasker program [6], they hit ~21 megabases of ERV sequence, or 2.0% of the genome. Together, the previously characterized and newly characterized ERVs represent more than 30 megabases of sequence and 2.9% of the chicken genome, a substantial increase over the previous figure of 1.3% of ERV sequences.

GGERV21, GGERV22 and GGERV30 are the most abundant lineages and account for more than half of all the viral sequences in the genome. However, only a few full-length elements were found for these abundant families; most of their sequences exist as fragments or solo LTRs. These abundant families are most closely related to the Birdawg and Kronos LTR elements previously identified as high copy number elements using cot-based sequencing and analysis of the chicken genome [18]. However, we did not identify any full-length elements corresponding to the Hithcock or Soprano LTR elements identified in the same study.

The LTRs at the 5' and 3' ends of a full-length ERV genomic sequence are generated from a single template during reverse transcription of RNA into DNA [19]. Therefore, at the moment that a full-length ERV integrates into the genome, its 5' and 3' LTRs are expected to be identical in sequence, and intra-element sequence differences between LTRs can be used to estimate the time that has elapsed since an element was active [20]. The ages of chicken ERVs were estimated in this way using the formula *t *= *d*/2*r*, where *t *is the time since insertion, *d *is the nucleotide sequence divergence per site between 5' and 3' LTRs of a single element and *r *is the rate of nucleotide substitution per site per million years. The value of *r *used here, 7.5 × 10^-4^, is based on comparisons of nuclear genes among four avian taxa [21].

Age ranges for the 11 novel ERV families we detected are shown in Figure 1. The youngest family of chicken ERVs is GGERV10, which includes 10 full-length elements with 5' and 3' LTRs that are either identical or differ by only 1 bp. The GGERV10 family of element sequences integrated from 0–3 million years ago. Full-length GGERV10 family members encode a ~1,600 base pair intact *gag *open reading frame (ORF) and a ~3,300 base pair *pol *ORF that encodes a polyprotein with homology to the protease, RT, RNAseH and integrase enzymes that catalyze reverse transcription. In other words, GGERV10 family members are potentially active ERVs that were integrated into the chicken genome very recently. Incidentally, the GGERV10 family is substantially younger than the GGERVLA (Figure 1) family that was previously described as the most recently active family in the genome [1].

The next youngest family is GGERV29, with elements that inserted 0–17.9 million years ago, and the oldest family we identified is GGERV24 at 136.5–143.9 million years old. This wide range of ages encompasses all newly discovered and previously characterized chicken ERVs. Even though the *ab initio *approach we used is best suited to find relatively young elements with readily identifiable structural elements (*i.e. *LTRs), it was able to detect families that were active hundreds-of-millions of years apart.

Using a combined evidence approach that integrates *ab initio *element detection with sequence similarity searches, motif identification and evaluation of element features we detected 11 novel ERV families covering more than 21 megabases of previously uncharacterized chicken genome sequence. Several of these families were fairly ancient, consistent with the expectation that degenerated element sequences may be missed by homology-based detection methods. However, a number of the ERVs we identified are members of young families that have been active very recently in the chicken genome. These results underscore the importance of integrating multiple methods [22] for the detection of interspersed repeats in eukaryotic genomes.

## Reviewers' comments

### Reviewer's report 1

Igor Zhulin, University of Tennessee and Oak Ridge National Laboratory

This is an interesting discovery of novel viral families in the chicken genome, which accounts for more than 2% of the genome sequence. I do not have any major concerns regarding this paper and support its publication; however, I would like to offer some comments for authors' consideration, mainly regarding the clarity and presentation.

### Authors' response

We are grateful to the reviewer Dr. Igor Zhulin for providing a number of very specific and constructive comments regarding the clarity and presentation of the manuscript. We revised the paper according to his suggestions.

### Reviewer's report 2

Itai Yanai, Harvard University

I support publication of this manuscript.

## Competing interests

The author(s) declare that they have no competing interests.

## Authors' contributions

AH and NP implemented LTR_STRUC on the chicken genome. AH performed all other sequence analyses and the phylogenetic analysis under the supervision of JFM and IKJ. AH and IKJ drafted the manuscript. All authors reviewed and approved the final version of the manuscript.
